# Progress in Medicine

**Published:** 1899-08-12

**Authors:** 


### Progress in Medicine.

### DISEASES OF THE BLOOD.

(*Continued from page* 316.)

**Hodgkin's Disease.**—Cases are not unknown in which this disease terminates as a leucæmia. Hence it is hardly logical to distinguish them as entirely distinct diseases, since, apart from the blood, the course is much the same in both. Harlow Brooks[6] records an instance of this kind where the final condition was that of lymphatic leucæmia, though at an early stage the leucocytes were normal in number and species. He would regard lympho-sarcoma as a more virulent variety of the same single disease, and he would apply Trousseau's name "Adeny" to all three states.

**Pernicious Anæmia.**—Engel[7] has found several cases in which Ehrlich's large nucleated embryonic cells were not present, and on the other hand he has discovered them in simple chlorosis. He regards the presence of megaloblasts as indicative rather of pathological changes in the red marrow than of actual pernicious anæmia. Of the latter macrocytes and metrocytes are specially distinctive. John S. Billings[8] insists that in severe cases of anæmia, from whatever cause, where the red corpuscles are less than 1,500,000, the absence of nucleated red cells, if search has been made through, say, ten specimens, is prognostic of a fatal result. They are, indeed, present in some fatal cases, but their presence shows the struggle of the organism to repair its injuries. Where there is no such effort he believes these cells do not appear, and he details the history of several cases in support of his theory. In the diagnosis of anæmias arising from intestinal hæmorrhages, W. G. Wyllie[9] advises that the patients be kept in bed for a week, and fed on peptonised milk and junket, while the bowels are opened freely by a mixture of castor oil and glycerine three times a day. This permits a minute examination of any tender spot in the abdomen, while any hæmorrhage is shown in the stools, which are otherwise yellow. Thus ulcers of the stomach duodenum and colon, appendicular or ovarian troubles are easily detected, and many cases of neurasthenia and severe anæmia are traced to small continuous hæmorrhages. A blackish stain, or a few drops of unaltered blood in the stools, will often thus reveal the cause of an obscure condition. Some cases of profound anæmia, again, depend on chronic septic infection through old salpingitis and ovaritis; and, finally, when much blood is lost in an operation he advises the instant restoration of the loss of fluid by saline enemata and injections. Southworth[10] considers that the prevalence of constipation in chlorotics does not warrant the belief in a fæcal anæmia. Moreover, urobilin, which is always increased where red cells are destroyed in large numbers, is actually decreased in the urine of chlorotics. As to treatment, rest in bed may be important; regulation of the diet and digestion should be provided for. The general preference at the present time is for large doses of the inorganic salts of iron. Naphthol and Blaud's pill form an excellent combination. Manges, in the same discussion, remarked that there is generally in chlorosis a hyperchlorydria, with normal motor power in the stomach, hence there is no objection to forced feeding in these cases, unless a gastric ulcer be present. Ph. Laurent praises a preparation of the oxalate of iron, combined with quassia, as free from the irritative effects of other forms upon the stomach and rectum. Eye symptoms in chlorosis are occasionally observed, and a case of retino-papillitis with violent headache and vomiting, is recorded by H. M. Bannister.[11] The patient was a young woman of 21. The headache came on suddenly, affected chiefly the right side of the head, and was accompanied by much photophobia. The optic neuritis was most marked in the left eye, and the vision was fairly good for near objects. Under treatment by iron and arsenic the patient made a rapid and complete recovery. If treatment is commenced soon enough restoration of sight is usually complete, but the author notices that in a case of Westcott's permanently impaired vision resulted. The pathology of these cases is obscure, and the condition has been ascribed by some writers to a form of auto-intoxication. A similar case, where the condition at first suggested brain tumour, was reported by H. T. Patrick at the same meeting of the American Neurological Association.

[6] The Post Graduate, Feb. [7] Brit. Med. Jour., Jan. 21. [8] New York Med. Jour., May 20. [9] Med. Rec., May 20. [10] Med. Rec., May 20. [11] Jour. of Neurology, p. 874, 1899.

